# Impact of Echocardiographic Cardiac Damage Staging on Mortality and Heart Failure Hospitalizations in Aortic Stenosis Patients Undergoing Transcatheter Aortic Valve Replacement

**DOI:** 10.3390/jcm14020408

**Published:** 2025-01-10

**Authors:** José A. Parada-Barcia, Manuel Barreiro-Pérez, José Antonio Baz, Rodrigo Estévez-Loureiro, Julio César Echarte, Victor Jiménez-Díaz, Andrés Íñiguez-Romo

**Affiliations:** Cardiology Department, University Hospital Alvaro Cunqueiro, 36312 Vigo, Spain; manuel.barreiro.perez@sergas.es (M.B.-P.); jose.antonio.baz.alonso@sergas.es (J.A.B.); rodrigo.estevez.loureiro@sergas.es (R.E.-L.); julio.cesar.echarte.morales@sergas.es (J.C.E.); victor.alfonso.jimenez.diaz@sergas.es (V.J.-D.); andres.iniguez.romo@sergas.es (A.Í.-R.)

**Keywords:** aortic stenosis, TAVR, echocardiography

## Abstract

**Background:** A significant proportion of elderly patients referred to transcatheter aortic valve replacement (TAVR) do not experience an improvement of their symptoms. New tools are needed to better select candidates and avoid futile procedures. The objective of this study was to evaluate the impact of a new echocardiographic classification which assesses the consequences of chronic elevation of afterload on mortality and hospitalizations for heart failure (HF) in patients with severe AS undergoing TAVR. **Methods:** This study included 130 high-risk, elderly patients with severe AS who underwent TAVR between January 2018 and December 2019. The patients were classified into three groups according to anatomical and functional features based on transthoracic echocardiography (TTE). The combined end point was death from all causes and HF admissions. **Results:** Echocardiographic staging was significantly associated with increased rates of death and HF hospitalizations. After multivariate adjustment, the patients with severe cardiac damage exhibited a significant increase in hospitalizations for HF and all-cause mortality (HR 4.79; 95% CI 2.00–11.05; *p* = 0.000), whereas the moderate cardiac damage group did not (HR 1.84; 95% CI 0.88–3.84; *p* = 0.104). **Conclusions:** Echocardiographic staging of severe AS could be a useful tool for predicting HF hospitalizations and all-cause mortality after TAVR in elderly, high-risk patients. Evaluating cardiac damage with this new score may be a promising strategy to better select patients and improve outcomes following TAVR.

## 1. Introduction

Severe aortic stenosis (AS) is a progressive and life-threatening condition that affects approximately 3% of adults over the age of 75 [1]. If left untreated, symptomatic severe AS is associated with a bleak prognosis [2]. Aortic valve replacement (AVR), either through surgical or transcatheter approaches, is currently the only treatment modality proven to improve survival and to provide long-term symptomatic relief [3]. Traditionally, surgical aortic valve replacement (SAVR) was the standard of care, but the advent of transcatheter aortic valve replacement (TAVR) has revolutionized the management of this condition. According to the current ESC/EACTS guidelines, transfemoral TAVR is recommended for older patients (≥75 years) or those who are at high risk (STS-PROM/EuroSCORE II >8%) or are unsuitable for surgery [4]. As the prevalence of AS continues to rise in tandem with an aging population, there is an increasing emphasis on refining patient selection criteria to ensure optimal outcomes. Identifying appropriate candidates is essential to maximize the benefits of the procedure while minimizing the risk of performing futile or unnecessary interventions. Recent evidence suggests that a substantial proportion of elderly TAVR candidates fail to experience significant symptomatic or functional benefits after the intervention, raising concerns about the adequacy of the current selection criteria [5]. In light of these challenges, there is a growing need for more refined tools to stratify patients and predict outcomes following TAVR. To address this gap, a novel staging system for severe AS was proposed. This classification is based on the extent of anatomic and functional cardiac damage evaluated by transthoracic echocardiography (TTE). The rationale behind this staging system is rooted in the observation that clinical outcomes in severe AS are not solely determined by the severity of the valvular obstruction, as measured by gradients or valve area, but are also significantly influenced by the downstream effects of chronic left ventricular pressure overload. Prolonged afterload elevation can lead to left ventricular hypertrophy, diastolic dysfunction, atrial enlargement, secondary mitral regurgitation, pulmonary hypertension, and right ventricular dysfunction. These sequelae collectively contribute to a more advanced disease state [6]. There is growing evidence in favor of using this classification, suggesting important prognostic implications [7]. However, the utility of this staging system in stratifying, specifically elderly, high-risk severe AS patients remains incompletely defined. The use of this classification in routine clinical practice is limited; therefore, a simplification of the classification may be adequate, as was previously proposed [8].

The aim of our study was to evaluate the prognostic impact of this novel staging system in a real-world cohort of high-risk patients with severe AS who have undergone TAVR.

## 2. Materials and Methods

### 2.1. Study Design and Participants

This is a retrospective observational study conducted on a cohort of 130 severe AS patients from the health area of Vigo, located in Galicia, Spain. All patients underwent TAVR between January 2018 and December 2019. These patients were identified through administrative databases, using the Galician Healthcare Service information system. Electronic medical records were analyzed to collect data on baseline clinical variables, TTE data (defined as the closest TTE to the TAVR procedure), and follow-up events. Echocardiographic measurements were performed by experienced physicians. All the data were collected, processed, and anonymized using a code. Follow-up was performed for the TAVR procedure between January 2018 and December 2019, and lasted until June 2022. Patients remained in the analysis until an event occurred or until the end of the follow-up.

### 2.2. Definitions

Severe AS was defined according to current guidelines as a mean aortic valve gradient ≥40 mm Hg and/or aortic valve area <1.0 cm^2^ (or an indexed aortic valve area < 0.6 cm^2^/m^2^) and/or a peak aortic jet velocity ≥4 m/s [4]. High-risk patients were defined through a Heart Team committee decision, involving cardiac surgeons, cardiologists, and a geriatric specialist. The surgical risk assessment included consideration of the EuroSCORE II, a risk model which estimates the 30-day operative mortality using an algorithm based on the presence of coexisting illnesses [9]. The presence and extent of extra-aortic valvular cardiac damage was evaluated using TTE. Generaux et al. proposed 5 stages of cardiac damage [6], which we integrated into three groups for the current study as follows: mild cardiac damage (stage 0: no signs of cardiac damage; stage 1: left ventricular (LV) damage (LV ejection fraction < 50%, LV mass index > 95 g/m^2^ for women or >115 g/m^2^ for men, or E/e′ > 14)), moderate cardiac damage (stage 2: mitral and/or left atrial (LA) damage (LA volume index > 34 mL/m^2^ or moderate mitral regurgitation grade ≥3 or presence of atrial fibrillation at the time of baseline echocardiography)), and severe cardiac damage (stage 3: pulmonary and/or tricuspid vascular damage (systolic pulmonary artery pressure ≥ 60 mmHg or tricuspid regurgitation grade ≥ 3)); and stage 4: right ventricular damage (tricuspid annular plane systolic excursion < 16 mm)) (see Graphical Abstract for more details). If more than 1 of the included criteria were present, the patients were hierarchically assigned to the highest (i.e., worst) group.

### 2.3. Outcomes

The primary outcome was a composite of the all-cause mortality and heart failure-related hospitalization that occurred between the TAVR procedure and the last follow-up.

### 2.4. Statistical Analysis

Baseline characteristics are described using frequencies and percentages for categorical data, and mean ± standard deviation for continuous data. The patients were divided according to cardiac damage category, as defined previously. The participant characteristics of the cardiac damage groups were compared using analysis of variance with multiple-testing correction (Bonferroni) for continuous variables or the chi-square test for categorical variables. The Kaplan–Meier method was used to calculate the survival and event rates for the different categories; a comparison of cumulative event rates between these groups was performed using the log-rank test. To evaluate the risk of all-cause mortality and HF admissions, a multivariable Cox proportional hazards analysis was performed. The multivariable adjustment was developed including all the variables with clinical significance and those that had been associated with a higher risk of the combined end-point in the univariate analysis (*p* ≤ 0.10). All statistical analyses were performed using Stata 16.1 (StataCorp, College Station, TX, USA). *p* < 0.05 was accepted as statistically significant.

### 2.5. Ethics

The entire data handling process complied with ethical and legal standards, particularly with the Declaration of Helsinki, and was approved by the local ethics committee of Hospital Alvaro Cunqueiro Comité de Investigación Pontevedra-Vigo-Ourense (approval code: HACACO-2024-1; registry 2024/259; approval date: 1 November 2024). Informed consent was not required for the present study.

## 3. Results

A total of 130 patients with severe AS were included and followed up. Sixty-seven patients (51.5%) were women; the mean age was 83.0 ± 5.1 years and 58 (44.6%) were in atrial fibrillation. The study population consisted of 53 patients (41%) with mild cardiac damage, 58 (45%) with moderate cardiac damage, and 19 patients (15%) with severe cardiac damage. The baseline characteristics are detailed in Table 1 and Table 2. The chronic kidney disease prevalence differed between the groups. There were no differences between the groups in the prevalence of other concomitant diseases, including dyslipidemia, chronic obstructive pulmonary disease, and hypertension. Ninety-four patients (72.3%) were treated with self-expandable valves. There were no differences between the groups in the rates of moderate to severe paravalvular leak, major vascular complications, and permanent pacemaker implantation.

**Figure 1:**
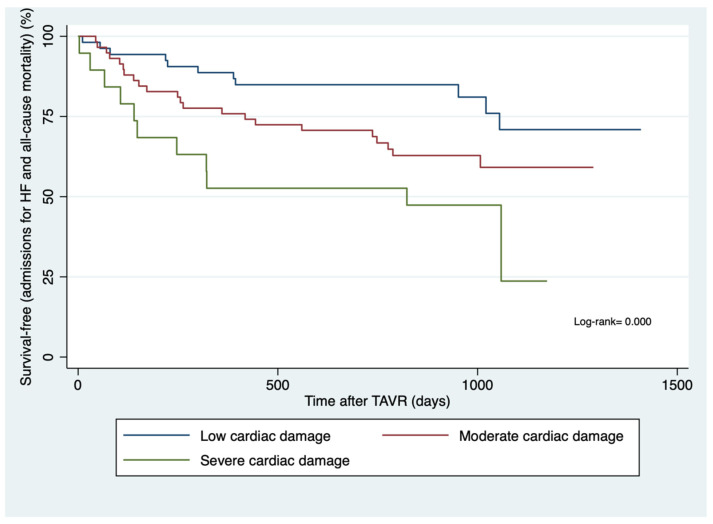
Incidence of all-cause mortality and hospitalizations for heart failure according to the echocardiographic cardiac damage stage.

During a mean follow-up of 2.26 ± 0.90 years, 5 (9%) mild cardiac damage patients, 12 (21%) moderate cardiac damage patients, and 7 (37%) severe cardiac damage patients were admitted for HF. Additionally, 9 (17%) mild cardiac damage, 17 (29%) moderate cardiac damage, and 8 (42%) severe cardiac damage patients died (Table 3). The incidence of HF and death increased as the cardiac damage progressed. The Kaplan–Meier survival curves for the primary endpoint by cardiac damage echocardiographic staging are presented in Figure 1.

To assess the impact of cardiac damage on the outcome, univariate and multivariate Cox regression analyses for the primary endpoint were performed (Table 4). After multivariate adjustment, the severe cardiac damage group showed a significant increase in hospitalizations for HF and all-cause mortality (HR: 4.21; 95% CI: 1.67–10.62), whereas the moderate cardiac damage group did not (HR: 1.18; 95% CI: 0.52–2.71).

## 4. Discussion

In this study of elderly and high-risk patients with AS treated with TAVR, we observed that an increased cardiac damage stage was associated with increased admissions for HF and all-cause mortality. Second, among our cohort, we detected a high prevalence of significant extra-valvular cardiac damage. These findings are likely to be relevant, as, despite the fact that an increasing number of patients are being treated with TAVR, tools to select those who may benefit most from TAVR have not been widely adopted. The current guidelines recommend the utilization of the traditional STS risk score for risk stratification to guide the decision between SAVR or TAVR; however, no specific statement has been made about how to predict better results after treatment to avoid futility.

Risk models perform best when developed from the population undergoing the specific procedure that is the focus of the model [10]. This is the reason why many efforts have been made to develop a specific risk model for patients undergoing TAVR. However, the first attempts showed subpar efficacy compared with the traditional STS score [11,12,13]. These scales included clinical information; however, they failed to include imaging-associated predictors. The echocardiographic staging system proposed by Généreux et al. was the first to introduce information derived from TTE, and it demonstrated competent performance in predicting 2-year mortality in comparison with STS scores [14]. In the original study of 1661 patients with a mean age of 73 years undergoing aortic valve replacement for severe AS, Généreux et al. found the stage of cardiac damage to be the strongest predictor of 1-year all-cause mortality, similar to what we observe in our analysis, confirming the impact of this classification in patient prognosis. The original study was performed in SAVR and TAVR patients, whilst our cohort did not include SAVR patients. Therefore, our cohort is older, with a mean age of 83 years, and has more comorbidities. Our findings are consistent with the recent study by Fukui et al., who conducted a study in a TAVR population and showed comparable results, despite an adjustment of the STS scores [15].

The utility of echocardiographic staging is consistent with previous reports, which showed that significant tricuspid regurgitation and right ventricle impairment were associated with a bleak prognosis after TAVR [16,17].

Furthermore, it was not surprising that in our population, BMI and peripheral artery disease were also independently associated with mortality, as major vascular complications are more prevalent in overweight patients [18]. The burden of comorbidities did not differ between groups and this emphasizes the importance of performing echocardiographic staging in our patients. Establishing a simplified staging classification using the 3-step stages presented in this paper and differentiating patients with isolated LV damage from those with damage extending to the mitral valve, and finally those with extent to the pulmonary circuit and right ventricle may help to extend the use of TAVR in clinical practice due to its simplicity.

From the 130 patients included in our study, 79 (59.2%) had at least moderate cardiac damage, a result which is similar to that reported by Fukui et al., and from this, the question arises whether early aortic valve intervention is adequate. In these cases, patients may possibly benefit from timely interventions when irreparable extra-valvular damage has not yet occurred, especially since there is evidence that LV and RV dysfunction can improve immediately after the relief of LV obstruction with TAVR. While the decision to operate on asymptomatic AS patients remains a matter of debate, recently, two randomized trials showed evidence in favor of early intervention. The Aortic Valve Replacement Versus Conservative Treatment in Asymptomatic Severe Aortic Stenosis (AVATAR) trial showed that the incidence of major cardiovascular events is lower with early surgery approaches compared to conservative approaches, although this study only included patients allocated to surgery [19]. The early TAVR group, likewise, contained asymptomatic patients with severe AS and their clinical parameters were compared to those of the early intervention group (ballon-expandable TAVR). Similar results supporting an early intervention were observed [20]. However, these findings only applied to the low-risk population in both trials. Therefore, further research is needed in high-risk patients.

Currently, there are no data available to guide the management and treatment of extra-valvular cardiac damage, either before or after TAVR. While medical therapy has proven to be ineffective in halting the progression of AS, it may hold promise in facilitating the recovery of extra-valvular cardiac damage following TAVR. Notably, a recent study by Paolisso et al. [21] demonstrated that the use of sodium–glucose cotransporter-2 inhibitors (SGLT2i’s) was associated with improved cardiac remodeling and a reduced risk of major adverse CV events at a 2-year follow-up in diabetic patients with severe AS and a left ventricular ejection fraction below 50%. The ongoing DapaTavi trial (NCT04696185) will provide further insights into the efficacy of SGLT2i’s in reducing extra-valvular cardiac damage, as this trial was designed to assess the efficacy and safety of the SGLT2i dapagliflozin in a diverse cohort of patients undergoing TAVR, particularly those at elevated risk of hospitalization due to heart failure [22].

## 5. Limitations

The main limitation of our study is that it was a retrospective, single-center study with a small population; however, most of the participants were at an advanced stage of disease, a finding which is relevant. Nevertheless, our study also has several strengths: recent studies have excluded more than 40% of the patients because of inadequate TTE information, a fact which may lead to some degree of selection bias [23]. In our study, echocardiographic imaging was performed with standard protocols and all patients had complete data to allow staging classification. It should be noted that mortality and worsening of HF in the current TAVR population, which is older and has many comorbidities, are multifactorial events that are not solely influenced by the extent of cardiac damage. However, we should take into account the big difference between the severe cardiac damage group and the other groups.

## 6. Conclusions

The echocardiographic staging of severe AS could be a simple and valuable aid for predicting HF hospitalizations and all-cause mortality after TAVR in elderly, high-risk patients. Evaluating cardiac damage with this new score may be a promising strategy to better select patients and improve outcomes following TAVR.

## Figures and Tables

**Table 1:** Baseline characteristics of the patients.

Variable	Mild Cardiac Damage (*n* = 53)	Moderate Cardiac Damage (*n* = 58)	Severe Cardiac Damage (*n* = 19)	*p* Value
Age, yrs	83.2 (6.3)	82.1 (5.7)	84.1 (5.1)	0.54
Male sex, No. (%)	27 (50.9)	32 (55.2)	9 (47.4)	0.81
BMI, No. (%)	27.6 (4.3)	28.1 (4.6)	26.6 (3.5)	0.43
Diabetes mellitus, No. (%)	15 (28.3)	28 (48.3)	6 (31.6)	0.08
Hypertension, No. (%)	37 (69.8)	49 (84.5)	15 (79.0)	0.18
Dyslipidemia, No. (%)	30 (56.6)	35 (61.4)	15 (79.0)	0.23
Euro-Score II	7.9 (3.6)	8.1 (3.9)	8.4 (3.4)	0.07
Glomerular filtration rate (mg/mL/min)	72.1 (21.7)	63.3 (21.4)	59.2 (19.5)	0.07
COPD, No. (%)	12 (22.6)	15 (25.9)	5 (26.3)	0.91
Peripheral arterial disease, No. (%)	8 (15.1)	11 (19.0)	2 (10.5)	0.66
Hemoglobin (g/dL)	11.7 (2.0)	11.9 (2.0)	11.6 (1.8)	0.81
Medium gradient, mmHg	46.3 (14.1)	38.7 (13.2)	42.7 (12.8)	0.01
AVA, cm^2^	0.76 (0.2)	0.76 (0.2)	0.68 (0.2)	0.28
Classical low-flow low-gradient AS, No. (%)	7 (14.9)	7 (14.3)	5 (27.8)	0.39
Prior pacemaker, No. (%)	5 (9.4)	9 (15.8)	1 (5.3)	0.38

AS: aortic stenosis; AVA: aortic valve area; BMI: body mass index; COPD: chronic obstructive pulmonary disease. Values are expressed as No. (%) or mean ± standard deviation.

**Table 2:** Procedural characteristics and in-hospital outcomes.

Variable	Mild Cardiac Damage (*n* = 53)	Moderate Cardiac Damage (*n* = 58)	Severe Cardiac Damage (*n* = 19)	*p* Value
BEV, No. (%)	14 (26.4)	14 (24.1)	8 (42.1)	0.30
In-hospital outcomes				
AKI (stage 2 or 3), No. (%)	8 (15.0)	12 (20.7)	6 (31.6)	0.30
Valve embolization, No. (%)	1 (1.9)	0	0	0.89
Moderate to severe PVL, No. (%)	9 (17.7)	9 (15.5)	0	0.17
Major vascular complications, No. (%)	5 (9.4)	7 (14.3)	2 (10.5)	0.41
Follow-up outcomes				
Myocardial infarction, No. (%)	1 (1.9)	0	0	0.48
PPI, No. (%)	14 (26.4)	15 (25.9)	4 (21.1)	0.89
Stroke, No. (%)	5 (9.4)	2 (3.5)	3 (15.8)	0.18
HF admissions, No. (%)	5 (9.4)	12 (20.7)	7 (36.8)	0.03
All-cause death, No. (%)	9 (17.0)	17 (29.3)	8 (42.1)	0.08
CV death, No. (%)	3 (5.7)	5 (8.6)	3 (15.8)	0.395

AKI: acute kidney injury; BEV: ballon-expandable valve; PPI: pacemaker implantation; PVL: paravalvular leak. Values are expressed as No. (%) or mean ± standard deviation.

**Table 3:** Follow-up events.

Variable	Mild Cardiac Damage (*n* = 53)	Moderate Cardiac Damage (*n* = 58)	Severe Cardiac Damage (*n* = 19)	*p* Value
Myocardial infarction, No. (%)	1 (1.9)	0	0	0.48
PPI, No. (%)	14 (26.4)	15 (25.9)	4 (21.1)	0.89
Stroke, No. (%)	5 (9.4)	2 (3.5)	3 (15.8)	0.18
HF admissions, No. (%)	5 (9.4)	12 (20.7)	7 (36.8)	0.03
All-cause death, No. (%)	9 (17.0)	17 (29.3)	8 (42.1)	0.08
CV death, No. (%)	3 (5.7)	5 (8.6)	3 (15.8)	0.395

CV: cardiovascular; HF: heart failure; PPI: pacemaker implantation. Values are expressed as No. (%) or mean ± standard deviation.

**Table 4:** Univariate and multivariate Cox analyses for the composite of all-cause mortality and rehospitalization for HF.

Variable	Univariate Analysis		Multivariate Analysis	
	Hazard Ratio (IC 95%)	*p* Value	Hazard Ratio	*p* Value
Low risk	Ref		Ref	
Intermediate risk	2.01 (0.98–4.16)	0.057	1.84 (0.88–3.84)	0.104
High risk	3.77 (1.63–8.70)	0.002	4.79 (2.00–11.5)	0
Age	0.95 (0.91–0.99)	0.049	0.96 (0.91–1.02)	0.173
Male sex	1.94 (1.05–3.59)	0.035	1.85 (0.96–3.58)	0.068
BMI	1.10 (1.02–1.17)	0.005	1.12 (1.05–1.21)	0.001
Peripheral arterial disease	2.34 (1.17–4.65)	0.016	2.14 (1.04–4.41)	0.038
Glomerular filtration rate	0.98 (0.97–0.99)	0.038	0.99 (0.98–1.03)	0.1
COPD	1.52 (0.81–2.88)	0.192		
Hypertension	1.51 (0.70–3.25)	0.292		
Classical low-flow low-gradient AS	0.74 (0.29–1.90)	0.536		
Prior pacemaker	1.20 (0.47–3.06)	0.696		
Prior stroke	1.59 (0.49–5.16)	0.436		

AS: aortic stenosis; BMI: body mass index; COPD: chronic obstructive pulmonary disease.

## Data Availability

Data are contained within the article; data can be shared if it is requested.
